# Childhood hypophosphatasia: to treat or not to treat

**DOI:** 10.1186/s13023-018-0866-7

**Published:** 2018-07-16

**Authors:** Eric T. Rush

**Affiliations:** 1 Division of Clinical Genetics, Department of Pediatrics, Children’s Mercy Kansas City, 2401 Gillham Road, Kansas City, MO 64108 USA; 2 Division of Endocrinology, Metabolism, and Genetics, Department of Internal Medicine, University of Kansas Medical Center, Kansas City, KS 66160 USA

**Keywords:** Hypophosphatasia, Alkaline phosphatase, Rare bone disease, Rickets, Brittle bone disease, Asfotase alfa, Enzyme replacement therapy

## Abstract

**Background:**

Hypophosphatasia (HPP) is a rare inborn error of metabolism that results from dysfunction of the tissue non-specific alkaline phosphatase enzyme. Its manifestations are extremely variable, ranging from early lethality to disease limited to the dentition. The disease is life-threatening when manifesting within the first six months of life, excepting the extremely rare benign perinatal hypophosphatasia. Childhood hypophosphatasia, defined as onset of symptoms between six months and eighteen years, can manifest as rickets, pain, decreased mobility, deficits of growth, and fractures. Historical treatment has generally involved a combination of dietary and rehabilitative interventions.

**Main document:**

Asfotase alfa (Strensiq™), is a first-in-class bone-targeted recombinant tissue nonspecific alkaline phosphatase which has shown significant improvements in morbidity and mortality in patients with perinatal and infantile hypophosphatasia. Subsequent research has also shown improvements in morbidity for patients with childhood hypophosphatasia as measured by improvement in rickets, growth, strength, mobility, and quality of life. This enzyme replacement therapy has generally been well-tolerated, with most adverse reactions being mild-to-moderate in nature.

The author shares their approach to decisions on commencement of ERT based from experience of managing approximately fifteen patients across the age spectrum. This approach focuses on assessing the severity of five key manifestations of childhood HPP: decreased mobility, pain, rickets, deficits of growth, and fractures.

## Background

Hypophosphatasia (HPP) is an inborn error of metabolism that is caused by mutations of the *ALPL* gene, which encodes the tissue nonspecific alkaline phosphatase (TNSALP) isoenzyme [1]. HPP is a rare and variable condition and presentation can occur at any time in the lifespan [2]. The incidence is not completely understood, but the incidence of 1 in 100,000 for severe disease based off of a population in Ontario [3] has been frequently cited. Estimates for less severe forms of hypophosphatasia have been made utilizing molecular data and have suggested an incidence of 1 in 6370 [4]. HPP has been divided into a number of subtypes based on clinical presentation and timing of onset and include perinatal-, infantile-, childhood-, and adult-onset. An additional category of odontohypophosphatasia is used for disease that limited to the teeth. HPP has classically been described as an autosomal recessive disorder like most other inborn errors of metabolism, but it is now known that it can be inherited in both an autosomal dominant or autosomal recessive manner. In one series of childhood HPP, 54% of patients had recessive disease, whereas 46% had dominant disease [5]. Although no reliable genotype/phenotype correlation has been described for specific variants [6], it is known that recessive disease tends to be more severe than dominant disease.

The clinical variability of HPP is considerable, with the majority of findings in HPP relating either directly or indirectly to effects of TNSALP dysfunction in bone [7]. Patients with perinatal or infantile HPP frequently manifest with severe deficit in bone mineralization, resulting in small thorax, pulmonary hypoplasia, and severe bowing of the limbs. Patients with childhood hypophosphatasia typically lack these life-threatening signs, but may still have significant disease, with poor mobility, chronic pain, and short stature [8]. These same patients may also have significant rickets, long bone deformity, and non-traumatic fractures [9]. Fractures which occur in patients with HPP may heal poorly and can reoccur [10].

Treatment of HPP has historically been supportive in nature, utilizing rehabilitative strategies to minimize functional limitations [11], surgery to manage some fractures, and in some cases modified diet to correct hypercalcemia [12]. More recently, enzyme replacement therapy (ERT) has become available for patients with HPP. This ERT, asfotase alfa (Strensiq™), is a first-in-class bone-targeted recombinant tissue nonspecific alkaline phosphatase that is administered subcutaneously by the patient or caregiver [13]. Significant evidence has been published in support of beneficial effects for survival and function in patients with perinatal and infantile HPP, and additional evidence has accumulated in support of improved function in patients with childhood HPP [14]. Appropriate selection of candidates for enzyme replacement therapy has continued to be challenging when patient do not have life-threatening disease. This author provides a discussion of the available data and how it has been utilized by a single clinician to make decisions to treat with ERT.

## Benefits of treatment

Patients with life-threatening HPP have experienced significant benefit from treatment with asfotase alfa. In 2012, a seminal article was published showing significant healing of rickets and improvement in ventilator status over 48 weeks of treatment [15]. Continued follow-up of this cohort showed continued improvement in skeletal mineralization and respiratory function [16–18]. These studies also reported that treatment was generally well-tolerated in those patients. Later comparison to historical controls showed a marked improvement in survival (95% versus 42% at one year, 84% versus 27% at five years) for patients with perinatal or infantile HPP. Importantly, 76% of patients who required ventilation survived on ERT, and of those patients 75% were able to be weaned from ventilation [19].

The spectrum of disease for HPP is much broader than these patients. Patients with childhood HPP typically survive, but many have chronic manifestations of disease. As previously noted, such manifestations may directly affect growth, mobility, and quality of life. Treatment therefore reflects this variation in disease. Research has been pursued according to these presumed goals of treatment. In childhood HPP, patients may suffer from rickets, fractures, hypotonia, short stature, or deficits in age-appropriate activities [20]. It has been noted that children between the ages of 5–12 had improvements in rickets as judged by the Radiographic Global Impression of Change (RGI-C) and the Rickets Severity Scale (RSS), evident within months. In the same cohort of patients, function improved as judged by increases in 6-min walk test (6MWT) and BOT-2 test of motor proficiency [21]. These improvements were observed to persist throughout the duration of the study. Additional evaluation of a similarly aged cohort of treated and control patients for mobility noted similar impairments at baseline followed by significantly greater rate of improvement in assessment scores for treated versus control patients [22]. Hypotonia is also a common concern in patients with childhood hypophosphatasia. Additional investigation has shown that patients had substantial muscle weakness and other physical limitations as compared to their peers, but after several years of treatment they experienced persistent improvement in muscle strength, agility, and pain [23, 24]. Craniosynostosis and abnormal dentition are also well-described features of hypophopsphatasia, but it is not known that therapy with asfotase alfa alters their natural history.

Asfotase alfa has been generally well-tolerated in the published studies. Injection site reactions and lipodystrophy have been commonly reported and have been generally of mild to moderate severity. Less commonly, severe injection site reactions, hypersensitivity, and ectopic calcifications have been reported [25]. The author’s experience is that mild and moderate skin injection site reactions and lipodystrophy are common and should be discussed as part of medication counselling prior to commencement of therapy. Although more severe hypersensitivity reactions are less common, they should still be discussed with patients and caregivers prior to starting therapy. Ectopic calcifications which have been reported have generally been asymptomatic and found in the conjunctiva or cornea. Lastly, although follow-up data from the previously discussed clinical trials continues to be presented, we still do not know what the longer-term effects of asfotase alfa therapy are for those patients. Patient registry data will be extremely helpful in learning more about these effects.

## Goals of therapy in childhood HPP

### Targets of therapy

Significant data has been accumulated related to treatment of patients with childhood HPP and suggests that treatment with ERT can improve outcomes, and provide ample evidence for selection of patients with life-threatening manifestations of HPP such as respiratory insufficiency or pyridoxine-responsive seizures. The available literature does not provide as much clarity on less severe patients, in whom the decision to treat is less clear. For those of us who routinely treat patients with ultrarare disease, this is familiar territory. For other clinicians less so, and all of us have had uncomfortable moments agonizing over a patient in whom the case for or against treatment may be legitimately made. The remainder of this article describes the framework used by the author in deciding which patients are appropriate for treatment and represents opinion. It is hoped that readers will find this opinion useful as a practical approach to patient care.

1. Mobility – Patients with childhood HPP may have limited mobility compared to their age-matched peers, and this may translate into delays in attainment of normal motor milestones [26]. Attainment of motor milestones shows reasonable correlation with severity of disease, but even patients with relatively normal motor milestones may have alterations in gait and stamina [8]. This may require patients to utilize assistive technologies to improve their ability for ambulation. For these patients, mobility concerns may represent a significant decrease in the quality of life. In patients who are not able to participate in age-appropriate activities or who do so at the expense of fatigue or pain that requiring protracted recovery period, additional treatment is indicated. For patients with HPP who have hypotonia, mobility limitations, or gait abnormalities focused physical therapy is indicated [11]. If such patient regain normal functional status after physical therapy alone, this may be sufficient treatment. However, in patients in whom rehabilitation alone does not result in significant restoration of function, use of enzyme replacement therapy should be considered.
2. Pain – Patients with childhood HPP also frequently complain of pain [27]. Patients may complain of joint or periarticular pain, bone pain, or muscular pain. For mild experience of pain, conservative treatment with physical therapy, rest, acetaminophen, and nonsteroidal anti-inflammatory drugs (NSAIDs) is reasonable and should be tried initially. However, in those patients whose pain is recalcitrant to a conservative approach, enzyme replacement therapy should be considered at least on a trial basis for 6 to 12 months to evaluate for efficacy. It is recommended that patients be followed several times during this trial with serial use of pain assessments such as the Wong-Baker scale to more objectively monitor response to therapy.
3. Rickets – The presence of rickets-like changes is a variable but well defined feature of childhood HPP and is a visible reflection of underlying defects in bone mineralization in children [28]. Rickets characteristically presents radiographically with metaphyseal widening and fraying, bowing of long bones, and generalized hypomineralization. The underlying pathology of rickets can continue into adulthood, and may be reflected on bone histomorphometry as osteomalacia [29]. These changes can predispose to fracture and impaired growth. The use of vitamin D and mineral supplementation is effective for other forms of rickets such as nutritional rickets or X-linked hypophosphatemic rickets. Unfortunately, these strategies have been largely ineffective for HPP [30]. ERT should be considered in patients with significant rickets which are contributing materially to symptomatology.
4. Growth – Short stature and failure to thrive have ben commonly described in patients with childhood HPP, and patients have frequently been described as “skinny”. When patients with childhood HPP were treated with ERT, they showed acceleration in both linear growth and weight gain. The same cohort of patients showed corresponding improvements in endurance, strength, and mobility [31]. It can be interpreted that height in many patients correlates to some degree with the overall amount of disability. Therefore, in the patient with normal height and good overall function, treatment with ERT would seem unnecessary. However, patients with short stature and poor function in whom HPP is attributed as causal for both, treatment with ERT would appear to be indicated. However, what about the patient with short stature without radiographic evidence of hypomineralization or rickets and who has good function? This becomes more problematic, as the risk of treatment even with a well-tolerated therapy may not be outweighed by significant benefit in this instance. In those instances, there may be benefit in looking at the patient’s growth velocity as a guide of past growth to gain insight into expectation of future growth. Some patients may have short stature and yet have a normal growth velocity. In such patients, a clinician could more easily make the case for conservative management and periodic follow-up. Conversely, a patient with poor growth velocity would appear to be at risk of future growth deceleration, which arguably constitutes its own functional consequence. Children with HPP may have short stature for additional reasons, and therefore if enzyme replacement therapy is being considered, it is appropriate to evaluate for other causes of short stature prior to treatment, particularly if those causes of short stature may be amenable to an alternate treatment.
5. Fracture – Hypophosphatasia is occasionally compared to osteogenesis imperfecta (OI) given the presence of bone fragility in both conditions. However, physiologically these conditions are quite different and the presentation is also frequently quite different. In conditions like osteogenesis imperfecta, fractures are frequent. This is true even in otherwise mild disease [32]. It is not clear that the incidence of fractures in patients with hypophosphatasia is as high as it is in osteogenesis imperfecta. This is an important distinction, patients with osteogenesis imperfecta are generally not treated until certain criteria such as recurrent fracture are met [33]. Since patients with hypophosphatasia fracture less frequently than patients with OI but experience different symptomatology, it would appear inappropriate to wait until a person with childhood HPP fractures to commence treatment. Therefore, while ERT should be strongly considered in cases of fracture, it should also be considered in the absence of fracture in a patient with significant functional limitations.

### How does this translate to patient selection?

In a patient with childhood hypophosphatasia who has normal development and no or minimal signs or symptoms of hypophosphatasia, the author would generally recommend conservative management with annual followup to assess for progression of disease. For patients who present with significant functional alterations, the author suggests that those be alterations be quantified by objective measures as much as possible such as video recorded 6-min walk test for later gait analysis, quality of life inventory such as the Pediatric Outcomes Data Outcomes Instrument (PODCI), Rickets Severity Scale (RSS), and objective pain assessment by Wong-Baker or similar tool. Physical Therapy referral evaluation of functional deficits is recommended regardless of ultimate decision to treat with enzyme replacement therapy. For those patients who have significant functional limitations in spite of conservative therapy, this author would suggest that enzyme replacement therapy should be strongly considered.

### Seeing HPP as an inborn error of metabolism

The approach to treating hypophosphatasia is in part informed by a physician’s experiences in treating other bone disorders. There are many different specialists who manage patients with rare bone disease, and each brings their unique expertise in the care of the patient. Although it is clearly appropriate to discuss HPP as rare bone disease, it is the author’s opinion that it should be understood and approached as we would any inborn error of metabolism for the purposes of management.

Recent literature has characterized HPP as a stable and chronic condition based on stability of deficits in height, weight, and bone mineral density in patients over time [34]. It was noted in this study that there was statistical stability for each group of HPP patients, but further evaluation of the literature suggests that while the group trends are correct as reported by the authors, there exists significant intergroup variability as evidenced by a number of outliers and reflected in statistics by very large standard deviations. Therefore, this author interprets the work in question as underscoring more unpredictability in the trajectory of patients with HPP than was suggested by the authors. This author’s experience suggests that some patients with previously mild disease may experience an increase in symptomatology with age and should be followed accordingly. As with other inborn errors of metabolism, the symptomatology should drive the decision to treat and that decision should be dynamic and should respond to progression of disease.

### Cost considerations

An impressive number of targeted treatments for orphan diseases have been developed since 2000, a number of which are also enzyme replacement therapy for inborn errors of metabolism [35]. That these medications are expensive on a per-patient basis is not in dispute. A detailed discussion of the economics of rare and ultra-rare diseases is outside of the scope of this manuscript, but clinicians should still endeavour to be good stewards of healthcare finances. In the opinion of the author, this is best served by careful patient selection for those patients who can benefit from treatment and weighing this against its potential risks.

## Conclusion

HPP is a rare and complex inborn error of metabolism, requiring the input of multiple providers for optimizing care. Ideally, the decision to treat will be made by clinicians who have extensive experience with this disease process, but the reality is that this is not always possible. For clinicians with less experience who are called to treat patients with HPP, this author would advocate for consultation with centers of excellence to discuss the risks and benefits of treatment prior to commencement.

HPP is also the last hereditary rickets disorder to have its own definitive treatment, which gives significant opportunity to explore which patients are most likely to benefit from enzyme replacement therapy. The label granted by most drug regulatory agencies is quite broad, and as such we as clinicians have a duty to ensure that therapy is utilized intelligently. It is the hope of the author that this article provides a single clinician’s experience in treating approximately twenty patients with hypophosphatasia and to spur discussion amongst clinicians to pursue best practices.

## Abbreviations

6MWT: Six minute walk test
ALPL: Alkaline phosphatase, liver/bone/kidney type
BOT-2: Bruininks-Oseretsky Test of Motor Proficiency, 2nd Edition
ERT: Enzyme replacement therapy
HPP: Hypophosphatasia
NSAID: Non-steroidal anti-inflammatory drug
RGI-C: Radiographic Global Impression of Change
RSS: Rickets severity scale
TNSALP: Tissue nonspecific alkaline phosphatase
